# Polymorphisms of a Collagen-Like Adhesin Contributes to *Legionella pneumophila* Adhesion, Biofilm Formation Capacity and Clinical Prevalence

**DOI:** 10.3389/fmicb.2019.00604

**Published:** 2019-04-05

**Authors:** Mena Abdel-Nour, Han Su, Carla Duncan, Shaopei Li, Deepa Raju, Feras Shamoun, Marine Valton, Christophe Ginevra, Sophie Jarraud, Cyril Guyard, Kagan Kerman, Mauricio R. Terebiznik

**Affiliations:** ^1^Ontario Agency for Health Protection and Promotion, Toronto, ON, Canada; ^2^Department of Laboratory Medicine and Pathobiology, University of Toronto, Toronto, ON, Canada; ^3^The Mount Sinai Hospital, Toronto, ON, Canada; ^4^Department of Physical & Environmental Sciences, University of Toronto Scarborough, Toronto, ON, Canada; ^5^Department of Biological Sciences, University of Toronto at Scarborough, Toronto, ON, Canada; ^6^Department of Cell and Systems Biology, University of Toronto at Scarborough, Toronto, ON, Canada; ^7^Polytech Clermont-Ferrand, Aubière, France; ^8^CIRI—International Center for Infectiology Research, Legionella Pathogenesis Team, Université de Lyon, Lyon, France; ^9^INSERM U1111, Lyon, France; ^10^Centre International de Recherche en Infectiologie, Claude Bernard University Lyon 1, Lyon, France; ^11^National Center for Legionella, Hospices Civils de Lyon, Lyon, France; ^12^BIOASTER Microbiology Technology Institute, Lyon, France

**Keywords:** collagen-like adhesion, biofilm, *Legionella pneumophila*, cell–cell adhesion, clinical prevalence, adhesion

## Abstract

Legionellosis is a severe respiratory illness caused by the inhalation of aerosolized water droplets contaminated with the opportunistic pathogen *Legionella pneumophila*. The ability of *L. pneumophila* to produce biofilms has been associated with its capacity to colonize and persist in human-made water reservoirs and distribution systems, which are the source of legionellosis outbreaks. Nevertheless, the factors that mediate *L. pneumophila* biofilm formation are largely unknown. In previous studies we reported that the adhesin *Legionella* collagen-like protein (Lcl), is required for auto-aggregation, attachment to multiple surfaces and the formation of biofilms. Lcl structure contains three distinguishable regions: An N-terminal region with a predicted signal sequence, a central region containing tandem collagen-like repeats (R-domain) and a C-terminal region (C-domain) with no significant homology to other known proteins. Lcl R-domain encodes tandem repeats of the collagenous tripeptide Gly-Xaa-Yaa (GXY), a motif that is key for the molecular organization of mammalian collagen and mediates the binding of collagenous proteins to different cellular and environmental ligands. Interestingly, Lcl is polymorphic in the number of GXY tandem repeats. In this study, we combined diverse biochemical, genetic, and cellular approaches to determine the role of Lcl domains and GXY repeats polymorphisms on the structural and functional properties of Lcl, as well as on bacterial attachment, aggregation and biofilm formation. Our results indicate that the R-domain is key for assembling Lcl collagenous triple-helices and has a more preponderate role over the C-domain in Lcl adhesin binding properties. We show that Lcl molecules oligomerize to form large supramolecular complexes to which both, R and C-domains are required. Furthermore, we found that the number of GXY tandem repeats encoded in Lcl R-domain correlates positively with the binding capabilities of Lcl and with the attachment and biofilm production capacity of *L. pneumophila* strains. Accordingly, the number of GXY tandem repeats in Lcl influences the clinical prevalence of *L. pneumophila* strains. Therefore, the number of Lcl tandem repeats could be considered as a potential predictor for virulence in *L. pneumophila* isolates.

## Introduction

*Legionella pneumophila* is an environmental bacteria and opportunistic pathogen that causes a severe form of pneumonia in humans, known as Legionellosis (Yu et al., 2002; Carratala et al., 2005; Santoro et al., 2007). This bacteria is ubiquitously found in freshwater bodies where it thrives in biofilms as an intracellular parasite in bacterivorous protozoa (Fliermans et al., 1981). However, outbreaks of legionellosis have been associated with *L. pneumophila* growing in human-made fresh water reservoirs, water-using devices and water distribution systems (Fisher-Hoch et al., 1981; Wadowsky et al., 1982; Stout et al., 1985). Infections occur when aerosols from contaminated water are inhaled into the respiratory cavity. In the lung *L. pneumophila* multiplies intracellularly in macrophages and epithelial cells to ultimately cause pneumonia in susceptible individuals, such as immunocompromised patients (Fields et al., 2002; Miller et al., 2012). Indeed, *L. pneumophila* infections are a major cause of hospital and community acquired pneumonia in industrialized countries (Yu et al., 2002; Carratala et al., 2005).

Bacterial attachment is essential for the multicellular behavior that leads to the colonization of environmental and organismal niches. The attachment to surfaces allows bacteria to escape mechanical clearance and allows for the formation of biofilms, which promotes resistance to antimicrobials and immune evasion, and consequently favors the formation and maintenance of infection foci (Colvin et al., 2011; Travier et al., 2013). Bacterial attachment is mediated by proteins known as adhesins. Adhesins are also crucial for bacterial aggregation and biofilm formation. Furthermore, adhesins expressed by pathogenic bacteria can bind to extracellular matrix (ECM) and host-cells surface receptors, which can lead to the co-opting of cellular functions and the internalization of the bacteria by host-cells (Pizarro-Cerda and Cossart, 2006; Lukomski et al., 2017).

To date, few *Legionella* adhesins and attachment mediators have been characterized for which little is known about their mechanisms of action (Lucas et al., 2006; Hoppe et al., 2017). Understanding their binding properties and mechanisms will hence provide valuable information to prevent the colonization of *L. pneumophila* in water systems and reduce the occurrence of legionellosis outbreaks. The *L. pneumophila* adhesin *Legionella* collagen-like protein (Lcl) binds to glycosaminoglycans (GAGs), heparin sulfate and fucoidan (Vandersmissen et al., 2010; Duncan et al., 2011). Collagenous proteins are highly evolutionarily conserved in their structure and function. There are over 18,000 different collagen-like proteins annotated in bacteria and more than 600 in viruses, in addition to those that are characterized in mammals (Xu et al., 2010). Collagen like proteins expressed by other pathogenic bacterial species are involved in binding to host surfaces, evasion of phagocytosis, invasion, and biofilm formation (Caswell et al., 2008a,b; Oliver-Kozup et al., 2011; Oliver-Kozup et al., 2013; Zhao et al., 2015). Accordingly, we have shown that Lcl facilitates the binding of *L. pneumophila* to biotic and abiotic surfaces and demonstrated that it plays a role in bacterial cell–cell attachment, aggregation, sedimentation and biofilm formation (Mallegol et al., 2012; Abdel-Nour et al., 2014). Thus, Lcl is likely involved in *L. pneumophila* colonization of environmental and respiratory niches (Vandersmissen et al., 2010; Duncan et al., 2011; Abdel-Nour et al., 2013b).

Structurally, Lcl contains three distinguishable regions: An N-terminal region with a predicted signal sequence, a central region containing tandem collagen-like repeats (R-domain) and a C-terminal region (C-domain) with no significant homology to known proteins (Duncan et al., 2011). We have reported that both the repeat and the C-terminal domain are essential for biofilm production although how exactly these domains are involved is unclear. Lcl R-domain contains tandem repeats of the collagenous tripeptide Gly-Xaa-Yaa (GXY). The GXY tripeptide is key for the molecular organization of mammalian collagen. It allows the trimerization of procollagen α-chains into triple-helical procollagen molecules and mediates the binding of collagenous proteins to different cellular and environmental ligands such as extracellular matrix (ECM) components, GAGs and nucleic acids (Yu et al., 2014; An et al., 2016).

Lcl, is polymorphic in the number of GXY tandem repeats encoded in its repeat domain (Pourcel et al., 2007; Vandersmissen et al., 2010; Duncan et al., 2011), a phenomenon that occurs in both clinical and environmental isolates of *L. pneumophila* serogroup 1. However, the effect of these polymorphisms on the functional properties of Lcl is unknown. In this study, we characterized the role of Lcl domains and GXY repeats polymorphisms on its structural and functional properties and their effect on the ability of *L. pneumophila* to bind to surfaces, produce biofilms and mediate cell–cell interactions.

## Results

### Lcl Forms Collagenous Triple Helix Structures

**Figure 1:**
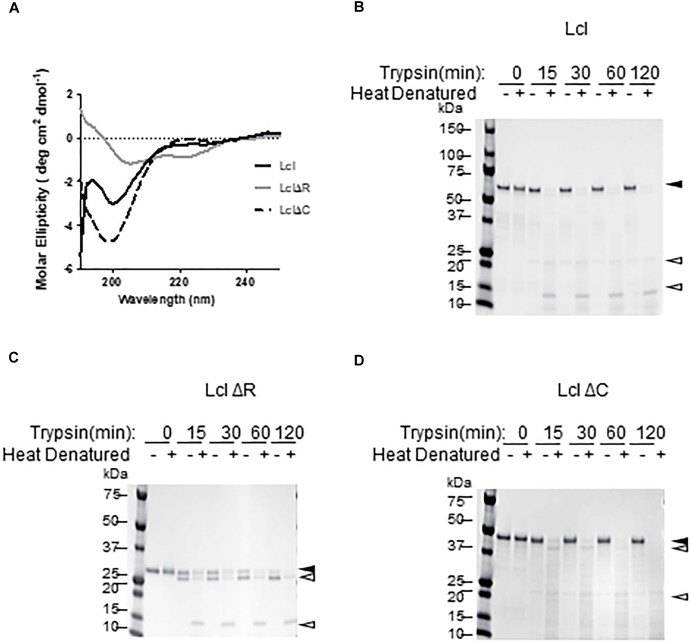
Lcl forms collagenous triple helix structures. **(A)** Circular dichroism profiles of full length His-tagged Lcl (Lcl), His-tagged Lcl lacking the C-terminal domain (LclΔC) and His-tagged Lcl lacking the collagenous repeat domain (LclΔR). **(B–D)** Coomassie stained gels of purified His-tagged Lcl **(B)**, His-tagged LclΔR **(C)**, and His-tagged LclΔC **(D)** after incubation with trypsin for the indicated time points with or without heat denaturing at 95°C for 10 min. Black arrowheads with labels indicated the full-length proteins and open arrowheads denote digestion products.

A hallmark of bacterial and eukaryotic collagenous proteins is the formation of triple-helix structures (Yu et al., 2014; An et al., 2016). To investigate if Lcl forms triple helices, we utilized circular dichroism (CD) to determine the secondary structure of his-tagged full length Lcl (Lcl) and truncated forms of the adhesin, lacking either the tandem repeats in the R-domain (LclΔR) or the C-domain (LclΔC). The CD spectra obtained for Lcl showed a wide negative ellipticity at 198 nm and a positive ellipticity at 220 nm, which is consistent with the spectral signature of a triple-helix secondary structure (Figure 1A), confirming the collagenous supersecondary structural nature of Lcl (Brodsky and Ramshaw, 1997). The triple helix structure is preserved in LclΔC, although with a more pronounced signal signature than the full-length protein. The CD spectrum for LclΔR, however, had a positive peak at 195 nm and two negative peaks at 204 and 220 nm, indicating that this mutant forms α-helix secondary structures but cannot assemble triple helix superstructures (Brodsky and Ramshaw, 1997). Therefore, the Lcl R-domain, containing GXY tandem repeats, is required for the formation of collagenous triple-helices structures. To validate this finding, we assessed the resistance of Lcl to trypsin digestion, as the formation of triple helices is known to confer collagenous proteins resistance to enzymatic hydrolysis by trypsin (Yu et al., 2014). In agreement with the structural analysis from the CD spectra, full length Lcl and LclΔC were resistant to trypsin, unless the triple helix structure was thermally denatured before the enzymatic treatment (Figure 1B–D). Collectively, these results indicate that Lcl forms triple-helices and that the R-domain is key for assembling this structure.

### Lcl Requires Both the R and C Domains to Bind to Fucoidan

**Figure 2:**
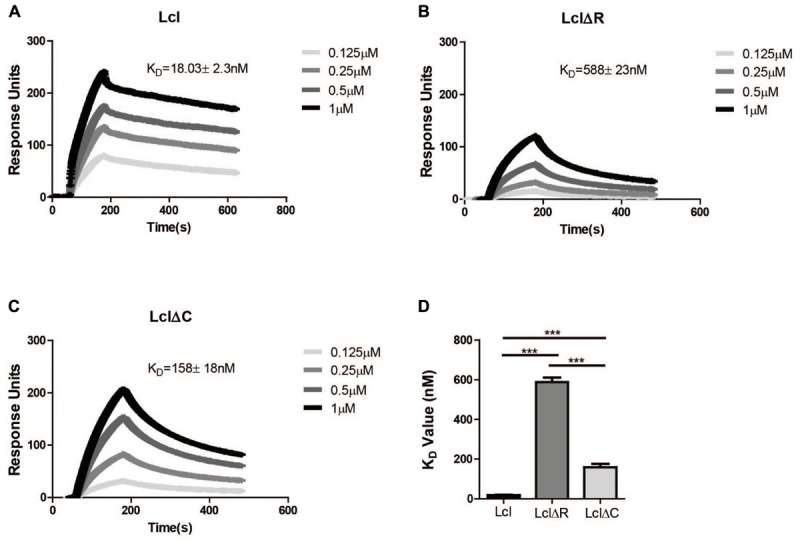
Both the R domain and C domain of Lcl are required for optimal binding to fucoidan. **(A–C)** Surface plasmon resonance sensorgrams of immobilized His-tagged purified Lcl **(A)**, His-tagged LclΔR **(B)**, and His-tagged LclΔC **(C)** to the indicated concentrations of fucoidan. **(D)** Bar graph of the dissociation constants of Lcl, LclΔR and LclΔC to fucoidan. The values plotted are the average of three individual experiments, ^∗∗∗^ denotes *p* < 0.001 by one-way ANOVA.

We have previously shown that Lcl is able to bind fucoidan, a polymer of fucose (Duncan et al., 2011). To assess the relative contribution of Lcl C and R-domains to the adhesin function of Lcl, we evaluated the binding affinity of LclΔC and LclΔR to fucoidan by surface plasmon resonance (SPR). In agreement with our previous reports, Lcl showed a strong affinity for fucoidan with a dissociation constant (KD) of 18 ± 2 nM (Figure 2A). However, the binding of both LclΔC and LclΔR were significantly reduced in comparison to the full-length molecule, with the binding of LclΔR being the weakest (*K*_D_ values of 158 ± 18 nM and 558 ± 23 nM, respectively) (Figure 2B–D). Taken together, our results demonstrate that both the R and C-domains contribute to the adhesin properties of Lcl, although the R-domain has a more crucial role in binding to fucoidan, presumably because of its role in the formation of the triple helix.

### Lcl Forms Supramolecular Complexes in a Manner Dependent on Both R and C Domains

**Figure 3:**
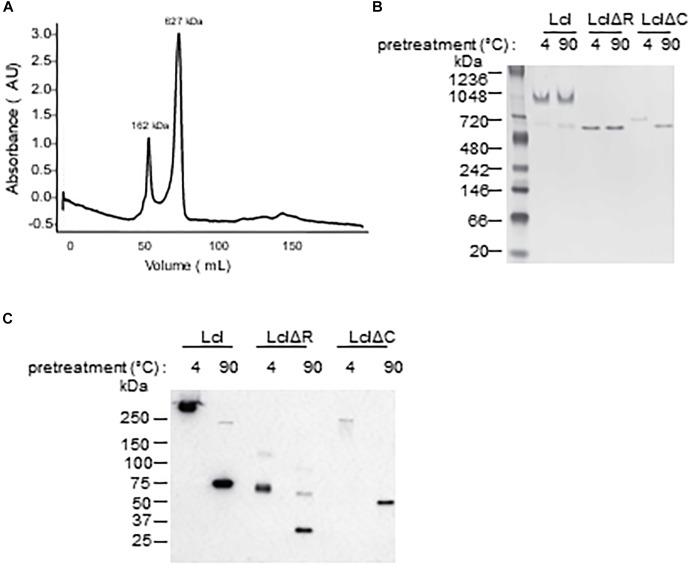
Lcl forms supramolecular complexes composed of trimers. **(A)** Size exclusion chromatography profiles of purified-His tagged Lcl **(A)**, values are the averages of 3 separate determinations and the molecular weights of standard proteins was estimated from the standard curve based on elution polymers. **(B,C)** His-tagged purified Lcl, LclΔR, and LclΔC was incubated at 4°C or at 90°C for 10 min and analyzed by Native-gel electrophoresis and visualized by Coomassie staining **(B)** and on SDS-PAGE and visualized by Western blotting using anti-His antibodies following treatment with Laemli buffer and boiling **(C)**.

**Figure 4:**
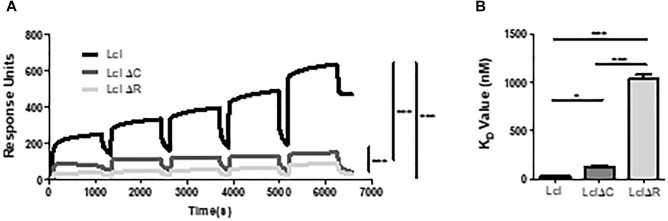
Lcl oligomerizes through homotypic binding of the R and C domains. **(A,B)** Surface plasmon resonance sensorgrams for single-cycle injection analysis of the interaction between His-tagged Lcl, His-tagged LclΔR and LclΔC against GST-tagged Lcl immobilized on anti-GST modified Biacore^TM^ sensor chips **(A)** and their associated calculated *K*_D_ values **(B)**. Plotted values are the average of 3 individual experiments, ^∗^ and^∗∗∗^ denotes *p* < 0.05 and *p* < 0.001, respectively, by one-way ANOVA.

Utilizing size exclusion chromatography, we detected the occurrence of Lcl triple helices with a molecular weight of 162 kDa, as well as the formation of larger molecular weight complexes of 627 kDa, which could be attributable to the oligomerization of Lcl trimers into supramolecular structures (Figure 3A). The large Lcl oligomers were also detectable in native PAGE and Western blot analysis. These complexes were resistant to heat denaturation (Figure 3B) unless treated with SDS for which the thermal denaturation yielded monomeric and trimeric Lcl (Figure 3C). LclΔR and LclΔC also formed supramolecular complexes. However, these structures were of significantly smaller size than those formed by the full-length protein, likely because of the smaller monomer molecular weight. We next investigated the contribution of R and C-domains to Lcl homotypic binding and determined the dissociation constants by SPR. The dissociation constants obtained showed that full length Lcl supramolecular complexes associate with high binding affinity (*K*_D_ of 28.7 ± 2.24 nM) to full length Lcl immobilized onto SPR sensor chips (Figure 4A). However, the binding affinity between Lcl supramolecular structures was significantly diminished in the case of LclΔC binding to immobilized full length Lcl (*K*_D_ = 123.4 ± 15.6 nM) and even much further reduced for LclΔR (*K*_D_ = 1037 ± 44.46 nM) (Figure 4B). Thus, Lcl forms oligomeric structures and both, R and C-domains are required for the effective oligomerization of the protein, to which they contribute independently, albeit the former playing a greater role in these processes.

### Biofilm Formation of *L. pneumophila* Clinical Isolates Is Associated With an Increased Number of Lcl Collagenous Repeats

**Figure 5:**
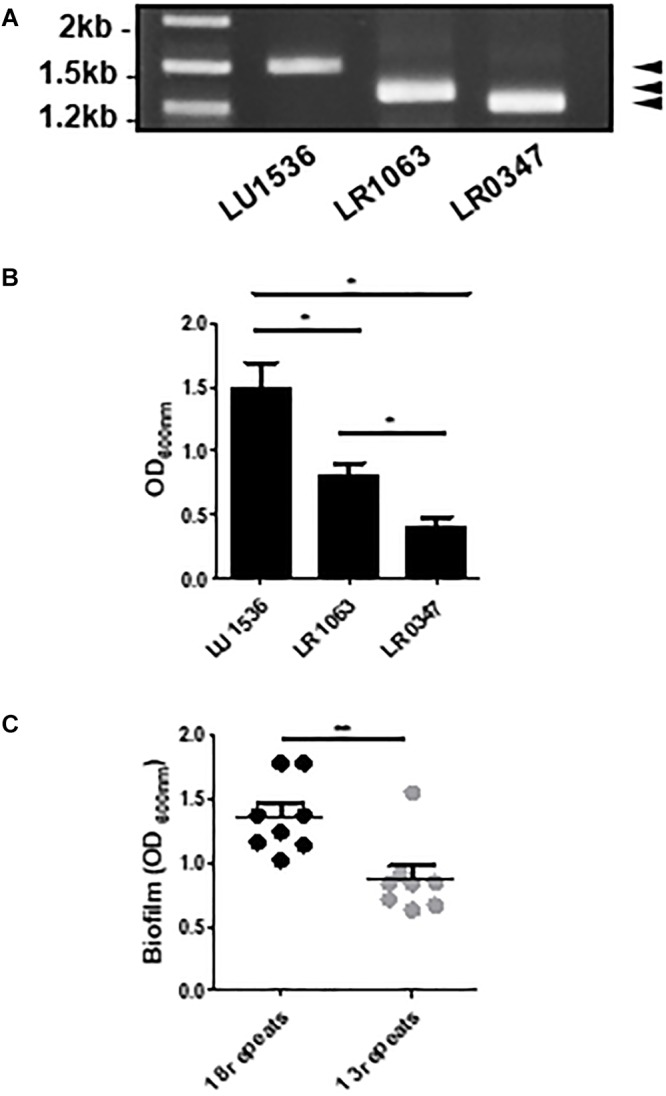
The number of Lcl collagenous repeats is correlated with biofilm production in *L. pneumophila* clinical isolates. **(A)** PCR amplification of *lcl* in the indicated *L. pneumophila* clinical isolates visualized by agarose gel electrophoresis. **(B)** Biofilm formation of 2 days old biofilms quantified by crystal violet of the indicated *L. pneumophila* clinical isolates. **(C)** Biofilm formation of 2 days old biofilms of *L. pneumophila* clinical isolates containing 18 and 13 repeats. Eight different strains were included per group and are indicated in Table 2. ^∗^ and^∗∗^ denotes *p* < 0.05 and *p* < 0.01, respectively, by one-way ANOVA.

Considering the effect of the R-domain on the structural and functional properties of Lcl, we next sought to investigate how Lcl polymorphisms affect the capacity of *L. pneumophila* to form biofilms. To assess this, we evaluated the performance of three clinical *L. pneumophila* serogroup 1 strains, LU1536, LR1063, and LR0347 that encode for *lcl* variants of approximately 1.5, 1.3, and 1.25 kb, respectively (Figure 5A). Their *lcl* PCR amplicons were sequenced revealing a 94.9–99.5% amino acid identity (Supplementary Table S1). In accordance with previously reported nomenclature, a single repeat was denoted as 5 Gly-Xaa-Yaa tripeptides (15 amino acids total) within the central tandem collagenous repeat domain (Vandersmissen et al., 2010). Using this designation, the predicted amino acid sequences of Lcl isoforms from LU1536, LR1063, and LR0347 contained 18, 13, and 11 repeat units, respectively (Supplementary Figure S1A). We next evaluated the ability of *L. pneumophila* serogroup 1 strains LU1536, LR1063, and LR0347 to produce biofilms. The biofilm production capacity of these strains positively correlated with the number of repeats in their respective Lcl proteins, while no significant differences were detected in bacterial growth (Figure 5B and Supplementary Figure S1B). When a pool of strains expressing Lcl of 18 and 13 repeats were analyzed, there was also a significant difference in biofilm formation (Figure 5C).

### Increased Number of Lcl Collagenous Repeats Favors *L. pneumophila* Sedimentation and Biofilm Formation

Strain intrinsic factors other than Lcl polymorphisms could account for differences in biofilm production by LU1536, LR1063, and LR0347. To evaluate whether Lcl polymorphisms play a significant role in *L. pneumophila* biofilm production levels, we measured the performance of their *lcl* homologs in an isogenic background. To this end, *lcl* genes were cloned into the *Legionella* expression vector pBH6119, under the control of the *icmR* promoter and subsequently transformed into a *lcl* knock out Lp02 strain, Lp02Δ*lcl* (Table 1). The newly generated complemented Lp02Δ*lcl* strains expressed Lcl proteins of the expected sizes and showed no significant differences in growth (Supplementary Figures S2A,B).

**Table 1:** *Legionella pneumophila* lab strains used in this study.

Species	Designation	Plasmid	Predicted Lcl size(s)	Source
*L. pneumophila*	Lp02	Untransformed	50 kDa	Renier et al., 2014
*L. pneumophila*	Lp02Δ*lcl*	Untransformed	–	Yu et al., 2014
*L. pneumophila*	Lp02 pBH6119	pBH6119	50 kDa	Yu et al., 2014
*L. pneumophila*	Lp02Δ*lcl* p	pBH6119	–	Yu et al., 2014
*L. pneumophila*	Lp02Δ*lcl* plcl 18rpts	p*lcl* 18rpts	50 kDa	This study
*L. pneumophila*	Lp02 p*lcl* 18rpts	p*lcl* 18rpts	50 kDa	This study
*L. pneumophila*	Lp02Δ*lcl* p*lcl* 13rpts	p*lcl* 13rpts	42 kDa	This study
*L. pneumophila*	Lp02 p*lcl* 13rpts	p*lcl* 13rpts	50 and42 kDa	This study
*L. pneumophila*	Lp02Δ*lcl* p*lcl* 11rpts	p*lcl* 11rpts	39 kDa	This study
*L. pneumophila*	Lp02 p*lcl* 11rpts	p*lcl* 11rpts	50 and 39 kDa	This study
*L. pneumophila*	Lp02Δ*lcl* p*lcl* Δrpts	p*lcl* Δrpts	25 kDa	An et al., 2016
*L. pneumophila*	Lp02 p*lcl* Δrpts	p*lcl* Δrpts	50 and 25 kDa	An et al., 2016

**Figure 6:**
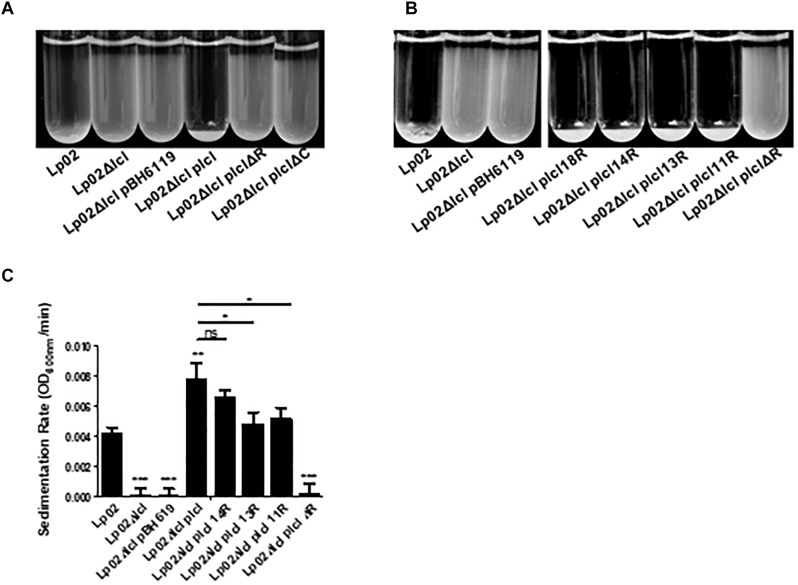
Both the R domain and the C terminal domain are required for *L. pneumophila* cell–cell binding. **(A,B)** Sedimentation assays of *L. pneumophila* in deionized water with 10% BYE after overnight incubation with Lp02Δ*lcl* transformed with p*lcl*ΔR and p*lcl*ΔC **(A)** and p*lcl* containing 18, 14, 13, 11 or Δ collagenous repeats **(B)**. **(C)** Sedimentation rates of Lp02Δ*lcl* transformed with p*lcl* containing the indicated numbers of collagenous repeats. Rates were calculated by calculating the rate of OD_600__nm_ decrease after the first 100 min. ^∗^, ^∗∗^, and ^∗∗∗^ denotes *p* < 0.05, 0.01, and 0.001, respectively, using one-way ANOVA between the indicated strains. Where no direct comparison is made symbols denote differences with Lp02. Experiments were performed in triplicate.

Lcl mediates cell–cell interactions that result in the auto-aggregation and sedimentation of *L. pneumophila* (Mallegol et al., 2012; Abdel-Nour et al., 2014), which ultimately leads to the formation of biofilms (Renier et al., 2014; He et al., 2016). As previously shown, the sedimentation of Lp02 requires *lcl* expression. Lp02Δ*lcl* sedimentation could be rescued by genetic complementation with the wild-type isoform of *lcl*, but not with *lclΔR and lclΔC* mutants (Figure 6A). The complementation of the Lp02Δ*lcl* strain with *lcl* isoforms of 18, 14, 13, or 11 repeats could also rescue sedimentation and, importantly, the sedimentation capacity of the chimeric strains showed a positive trend between the number of tandem repeats encoded in the *lcl* alleles (Figure 6B,C).

**Figure 7:**
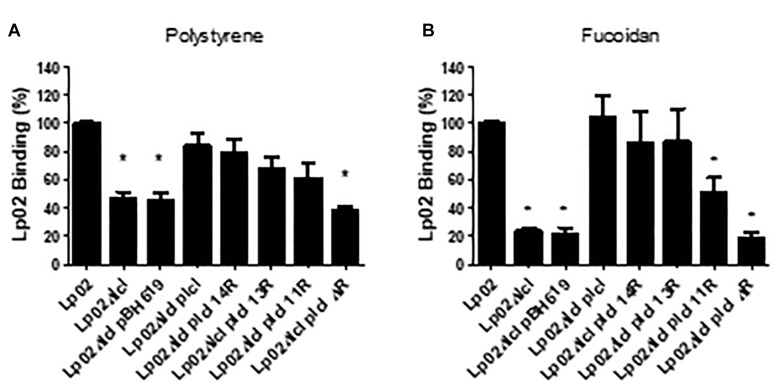
Increased number of repeats in the R domain promote stronger binding of *L. pneumophila* to surfaces. **(A,B)** The binding of the indicated *L. pneumophila* strains measured by RT-qPCR after 1 h of attachment to polystyrene 96-well plates **(A)** or 96-well plates coated fucoidan **(B)**. ^∗^ denotes *p* < 0.05 by one-way ANOVA. Experiments were performed in triplicate.

The attachment of Lp02 to polystyrene and to fucoidan-coated surfaces was strongly dependent on Lcl, (Figure 7A,B) and similar to what was observed for bacterial sedimentation, the binding of Lp02Δ*lcl* to surfaces was effectively rescued by genetic complementation with *lcl* variants of 18, 14, 13, or 11 repeats. Importantly, the binding of the Lp02Δ*lcl* complemented strains to polystyrene and to fucoidan-coated surfaces showed a positive trend with the number of tandem repeats expressed by the Lcl variants (*r*^2^ = 0.8403 for polystyrene, *r*^2^ = 0.8084 for polystyrene) (Figure 7A,B).

**Figure 8:**
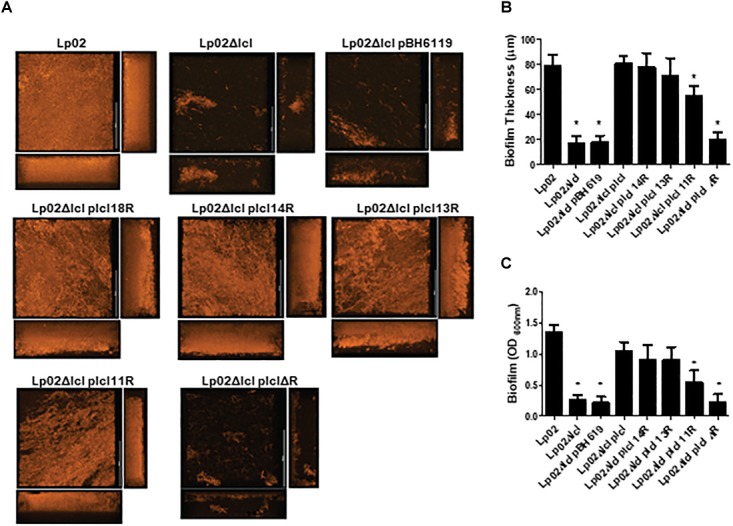
*Legionella pneumophila* biofilm production and thickness is positively correlated with the number of Lcl repeats. **(A,B)** Representative confocal laser scanning microscopy (CLSM) images **(A)** and thickness quantification **(B)** of 2 days old *L. pneumophila* biofilms of the indicated strains. **(C)** Biomass of 2 days old biofilms of the denoted *L. pneumophila* strains measured by crystal violet. ^∗^ denotes *p* < 0.05 between the indicated strain and Lp02 by one-way ANOVA. Experiments were performed in triplicate.

We next hypothesized that polymorphisms in Lcl collagenous tandem repeats may influence the capacity of Lp02 strains to form biofilms. Indeed, biofilm development, assessed by confocal microscopy revealed that Lp02Δ*lcl* complemented with *lcl* variants with 18,14, 13, and 11 repeats produced abundant biofilm structures (Figure 8A), which was not the case for Lp02Δ*lcl* p*lclΔR* that formed only scattered bacterial aggregates. The thickness of the biofilm structures, measured in 3D projections rendered from confocal imaging data, correlated positively with the number of tandem repeats in *lcl* (*r*^2^ = 0.7477, Figure 8B). This was further confirmed by quantifying biofilm development with crystal violet (O’Toole, 2011) (Figure 8C). Therefore, our results confirm that the repeat domain of Lcl is required and necessary for the formation *L. pneumophila* biofilms and demonstrates that the ability of Lcl to mediate biofilm formation increases with the number of tandem repeats.

### Lcl Collagenous Repeats Promote Oligomerization and Binding to Fucoidan

**Figure 9:**
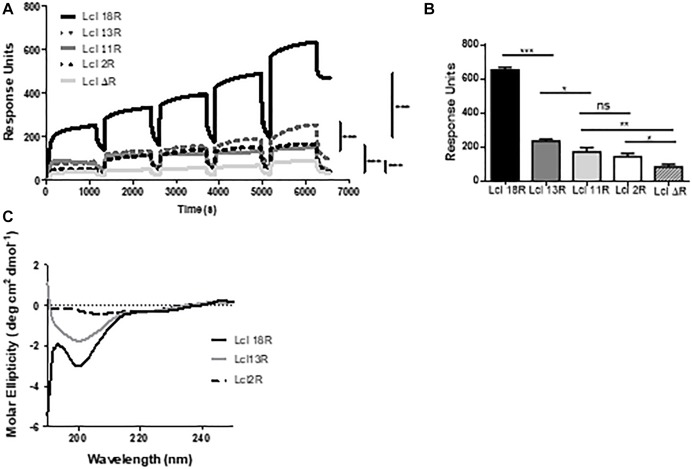
The binding affinity of Lcl increases with the number of collagenous repeats. **(A,B)** Surface plasmon resonance sensorgrams for single-cycle injection analysis of the interaction between His-tagged Lcl with 18 repeats (Lcl 18R), His-tagged Lcl with 13 repeats (Lcl 13R), His-tagged Lcl with 2 repeats (Lcl 2R), and His-tagged Lcl with 0 repeats (Lcl ΔR) against GST-tagged Lcl immobilized on anti-GST modified Biacore^TM^ sensor chips. **(B)** Calculated *K*_D_ between His-tagged Lcl 18R, His-tagged Lcl 13R, His-tagged Lcl 11R, His-tagged Lcl 2R, and His-tagged Lcl ΔR measured by Surface plasmon resonance to fucoidan at a concentration of 500 μM. **(C)** Circular dichroism profiles of Lcl 18R, Lcl 13R, Lcl 2R, and Lcl ΔR. Plotted values are the average of 3 individual experiments. ^∗^, ^∗∗^, and ^∗∗∗^ denotes *p* < 0.05, 0.01, and 0.001, respectively, using one-way ANOVA. Experiments were performed in triplicate.

Considering the effect that the number of Lcl GXY tandem repeats has on Lp02 biofilm formation capacity, we next sought to investigate the effect of these polymorphisms on the functional and structural properties of Lcl. To this end, we first determined the binding affinity of Lcl variants to wild type recombinant Lcl by SPR analysis. As shown in Figure 9A, the binding affinity of Lcl with 18, 13, and 11 repeats to recombinant Lcl (18 repeats) immobilized on the SPR sensor, increases with the number of repeats. Similarly, the binding affinity of Lcl polymorphic variants to fucoidan, determined by SPR, also followed a positive trend with the number of tandem repeats expressed in the Lcl molecules (Figure 9B). Accordingly, the CD spectra indicates that the magnitude of the triple-helix signature increases with the number of tandem repeats in the R-domains of Lcl variants (Figure 9C). Taken together these results suggest that the number of GXY tandem repeats in Lcl R-domain correlates positively with the functional properties of Lcl, increasing its binding affinity to carbohydrates and the homotypic binding that leads to the formation of supramolecular aggregates.

### Impact of the Number of Lcl Collagenous Repeats on the Clinical Prevalence of *L. pneumophila* Strains

**Figure 10:**
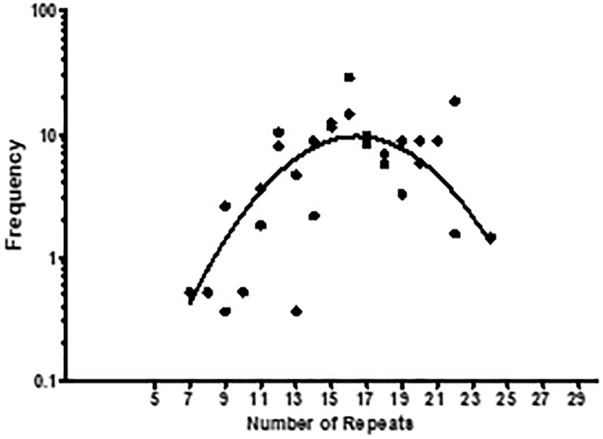
The number of Lcl collagenous GXY repeats influences the clinical incidence of *L. pneumophila* strains. Clinical strains from Public Health Ontario and the French National Reference Center for *Legionella* repositories were characterized respect to the number of GXY repeats encoded in their *lcl* genes. The frequency of the strains was then calculated by summing the number of strains encoding a given *lcl* gene variant, divided by the total number of strains in both repositories. This was plotted as a function of the number of *Lcl* GXY repeats. A total of 467 clinical isolates were tested.

Considering that the number of collagenous repeats determines the capacity of Lcl binding and Lp02 biofilm formation, we next asked if Lcl polymorphisms could determine the clinical incidence of *L. pneumophila* strains. To investigate this, we took advantage of two repositories of *L. pneumophila* clinical strains from Public Health Ontario (Tijet et al., 2010) and the French National Reference Center for *Legionella* (Cassier et al., 2015). A total of 467 clinical isolates were characterized with respect to their number of *lcl* repeats, determined by PCR amplification of the repeat domain in the *lcl* gene. We next plotted the frequency of occurrence of these isolates against the number of GXY repeats in their *lcl* gene (Figure 10). The data we obtained could be fitted with a Gaussian curve (*r*^2^ = 0.5154) that showed a maximal clinical incidence for strains with *lcl* genes encoding 16 GXY repeats. Importantly, the first phase of this curve (5–18 repeats) indicates that the number of GXY repeats in Lcl, contributes positively to the clinical incidence of *L. pneumophila*. These results may be attributed to the enhanced binding and biofilm-forming capacity of *L. pneumophila* that increases with the number Lcl GXY repeats. These qualities may result in greater persistence of the *L. pneumophila* in the sources of outbreaks and/or improve the bacterial capacity to establish infection foci in the lung (Abu Khweek and Amer, 2018). However, the decay in the incidence of clinical strains showed in Figure 10 for GXY repeats numbers larger than 18, may also indicate that after a certain threshold, larger Lcl binding capacities become detrimental for the persistence of *L. pneumophila* in the source of infection or during the infection process. It could be speculated that a more robust biofilm or the formation of larger bacteria aggregates, corresponding with a larger number of GXY repeats, may compromise the aerosolization of the bacteria cells that is required for its inhalation into the lungs.

## Discussion

We have previously described the role of Lcl in facilitating the binding of *L. pneumophila* to surfaces and demonstrated that this adhesin plays a role in bacterial cell–cell attachment, aggregation, sedimentation and biofilm formation. Thus, Lcl is likely involved in *L. pneumophila* colonization of environmental niches and facilitating human infections (Vandersmissen et al., 2010; Duncan et al., 2011; Mallegol et al., 2012; Abdel-Nour et al., 2013b, 2014). In this study we evaluated the contribution of both Lcl R and C terminal domains, to the structure and functional properties of this adhesin, and determined the impact of R domain polymorphisms on these phenotypes.

Many structural and functional characteristics of bacterial and eukaryotic collagenous proteins depend on the formation triple-helix structures (Yu et al., 2014; An et al., 2016). The formation of such triple-helices first requires the trimerization of the single polypeptides, which are mediated by ancillary coiled coil or globular domains (Bourhis et al., 2012). The tandem repeats of the collagenous Gly-Xaa-Yaa (GXY) tripeptide, facilitate trimers of procollagen α-chains to form triple-helix structures. Furthermore, the triple helix structure mediates the binding of collagenous protein to different biological and environmental ligands such as ECM components, GAGs and nucleic acids (Yu et al., 2014; An et al., 2016). Accordingly, our CD spectra and trypsin proteolysis assays on full length and delta R Lcl, demonstrated that Lcl forms triple helix structures that depend on the GXY tripeptide repeats in the R-domain and hence confirmed the collagenous nature of the molecule, predicted from the analysis of its sequence.

Assessment of the binding affinity of full length Lcl and Lcl mutants to fucoidan, yielded dissociation constants that confirmed the semiquantitative binding data we have previously reported utilizing ELISA-like assays. Importantly, although both R and C-domains are required for Lcl binding to fucoidan, the high *K*_D_ values for the ΔR Lcl mutant proves the strong dependence of Lcl binding properties on the GXY repeats and therefore in the formation of triple helix structures, similar to observations reported for Scl1 and Scl2 adhesin proteins of *Streptococcus* (Xu et al., 2002).

Unlike mammalian collagens, bacterial collagen-like proteins do not typically form fibrils and lack the modified amino acid hydroxyproline which promotes stability of the triple helix through stereoelectronic effects seen in type 1 mammalian collagen, with the exception of collagen-like proteins produced by *Bacillus anthracis* and *Streptococcus pyogenes* (Bella et al., 1994; Shoulders and Raines, 2009; Yu et al., 2014; Schnicker and Dey, 2016). Although, we could not detect the formation of fibrils by Lcl, as per transmission electron microscopy (data not shown), three different analytical approaches, size exclusion chromatography, electrophoresis and SPR proved that Lcl oligomerizes to form large supramolecular aggregates. The formation of such supramolecular structures has been associated with the functionality of different adhesin molecules (Roggenkamp et al., 2003; Nithianantham et al., 2009; Schroeder et al., 2009; Temoin et al., 2012). Thus, multivalent Lcl oligomers may show higher substrate avidity than the monomeric form of the adhesin. This suggests that oligomeric Lcl is therefore more efficient in crosslinking bacteria as well as mediating its attachment to surfaces, leading to more efficient biofilm formation. Our data indicates that both C and R-domains are required for the formation of the large supramolecular aggregates, although the R-domain plays a more prevailing role. It is therefore tempting to suggest that the main role performed by the R-domain is actually to allow for the formation of a more effective binding complex. Indeed, there are multiple examples of tandem repeats in adhesin proteins influencing bacterial host cell adhesion (Levdansky et al., 2010; Sheets and St Geme, 2011) binding specificity (Oh et al., 2005) and cell surface positioning of ligand binding domains (Hartford et al., 1997; Sylvestre et al., 2003).

The analysis of the collagenous domains of Lcl, from different *L. pneumophila* isolates, revealed polymorphisms in the numbers of GXY repeats in their R-domain (Vandersmissen et al., 2010; Duncan et al., 2011). Intriguingly, we found that number of GXY repeats in Lcl variants, correlates positively with the intensity of the triple-helix signature and the functional properties of this adhesin, which in turn determines *L. pneumophila* aggregation, surface adherence and biofilm forming capacity.

Polymorphisms in the numbers of GXY repeats has been also described for other bacterial collagen-like proteins (Paterson et al., 2008; Oliver-Kozup et al., 2011). In correspondence with our findings, the numbers of GXY repeats has been reported to affect the magnitude and stability of the triple-helix domain in engineered collagenous proteins (Han et al., 2006; Kar et al., 2006; Yu et al., 2011) and *Bacillus amyloliquefaciens* species with increased number of GXY repeats in their adhesins have both, more robust biofilms and faster sedimentation (Zhao et al., 2015).

The biofilm forming capacity of *L. pneumophila* depends on the expression of Lcl and here we demonstrated that biofilm formation increases with the number of GXY repeats encoded in the adhesin. Thus, the capacity of given *Legionella* strains to colonize environments may vary according to the number of GXY repeats in their Lcl proteins.

Numerous reports have associated legionellosis outbreaks with the occurrence of biofilms at the sources of infections (Abdel-Nour et al., 2013a; Bedard et al., 2016). Biofilms may allow *L. pneumophila* to persist in man-made water reservoirs and distribution systems, increasing the chances of aerosolization and infection. This agrees with the herein reported positive impact that GXY repeats has on the clinical incidence of *L. pneumophila* strains. Indeed, strains expressing Lcl variants encoding between 15 and 18 GXY repeats produce more robust biofilms than those observed for strains carrying Lcl variants with lower numbers of GXY repeats. In this regard, *L. pneumophila* Lp02, which is derived from the causative agent of the 1976 outbreak in Philadelphia, expresses a Lcl variant with 18 repeats. However, strains with greater than 18 repeats showed a reduced clinical incidence. This could be explained by several phenomena. It is possible that the increased binding affinity of Lcl with greater than 18 GXY repeats, may promote the formation of larger bacteria aggregates and denser biofilm structures with a reduced capacity for the diffusion of nutrients and hence less suitable for the colonization of oligotrophic human-made water reservoirs, considered to be the main source of legionellosis outbreaks. Stronger bacterial aggregates and biofilm structures may also be deficient in shedding bacterial cells for its aerosolization and thereby reducing the changes of inhalation an infection in humans. Additionally, as Lcl is immunogenic (Khan et al., 2013), it could be speculated that higher numbers of repeats while promoting Lcl oligomerization may also increase the antigenicity of Lcl triggering a more efficient immune response against the infection.

Our results indicate that the GXY repeats are crucial for maintaining the collagen-like structure and the functional properties of Lcl, in addition to conferring the capacity of the bacteria to attach to and form biofilms. Furthermore, we have shown that the number of Lcl GXY repeats can strongly influence the persistence and infectivity of clinical isolates, hence making Lcl polymorphisms a useful tool to predict virulent strains of *L. pneumophila* and potential sources of human infection.

## Materials and Methods

### Chemicals, Bacterial Strains and Growth Conditions

Unless otherwise indicated, all chemicals were purchased from Sigma. All *Legionella pneumophila* isolates were cultured in buffered charcoal-yeast extract (BCYE) agar at 37°C and 5% CO_2_ and or with buffered yeast extract (BYE) broth at 37°C with shaking at 100 rpm (Feeley et al., 1979). Cultures of Lp02 were supplemented with thymidine when required (Berger and Isberg, 1993). The list of *L. pneumophila* strains used is found in Table 1. Clinical isolates of *L. pneumophila* which were used in biofilm assays in Figure 4, are found in Table 2.

**Table 2:** *Legionella pneumophila* clinical strains used in this study.

Species	Designation	Number of Lcl Repeats	Predicted Lcl size(s)	Source
*L. pneumophila*	LU1536	18	50 kDa	Yu et al., 2014
*L. pneumophila*	LR1063	13	42 kDa	Yu et al., 2014
*L. pneumophila*	LR0347	11	39 kDa	Yu et al., 2014
*L. pneumophila*	LR3028	18	50 kDa	This study
*L. pneumophila*	LR0416	18	50 kDa	This study
*L. pneumophila*	LR4098	18	50 kDa	This study
*L. pneumophila*	LR3464	18	50 kDa	This study
*L. pneumophila*	LR4037	18	50 kDa	This study
*L. pneumophila*	LR3028	18	50 kDa	This study
*L. pneumophila*	LR4098	18	50 kDa	This study
*L. pneumophila*	LR1155	13	42 kDa	This study
*L. pneumophila*	LU1372	13	42 kDa	This study
*L. pneumophila*	LU2257	13	42 kDa	This study
*L. pneumophila*	LR1178	13	42 kDa	This study
*L. pneumophila*	LR0625	13	42 kDa	This study
*L. pneumophila*	LR2445	13	42 kDa	This study
*L. pneumophila*	LR0482	13	42 kDa	This study

### General DNA Techniques

Genomic DNA and plasmid DNA was purified using a QIAamp DNA minikit and a QIA prep spin miniprep kit (Qiagen) respectively. To quantify DNA, spectrophotometry was used. For PCR, 10 ng was used as a template and PCR reactions were performed with Taq DNA polymerase as recommended by the manufacturer (Invitrogen). PCR reactions for cloning were performed with Platinum Taq DNA polymerase high fidelity as per the manufacturer (Invitrogen). All clones were verified by sequencing. Sequencing reactions were performed using a BigDye terminator cycle sequencing kit, version 3.1 and purified with a BigDye X terminator purification kit and run on a 3130xl genetic analyzer (Applied Biosystems).

### Production and Purification of His-Tag Fusion Proteins

The *lcl* gene was amplified from LU1536, LR1063 and LR0347 genomic DNA using primers 3 and 4 (Table 2). To obtain an *lcl* gene with 2 repeats, a gene was designed with two repeats from sequences that were conserved amongst all the isolates used. This sequence was then synthesized (Genscript) and put into the pUC57 vector flanked with EcoR1 and Xho1 restriction sites and PCR amplified using primers 8 and 9 (Table 2). The PCR products were cloned into the pBAD-HisB (Invitrogen) vector according to the instructions of the manufacturer and cloned into the *Escherichia coli* LMG194 strain. *E. coli* LMG194 clones were tested for the expression of recombinant proteins after induction with 0.002% to 0.2% L-arabinose at 37°C for 4 h and the optimal arabinose concentration for maximum expression was obtained and used for purification. His-tagged fusion proteins were purified under native conditions with a nickel-Sepharose high-performance chromatography column (HisTrap HP column) according to the instructions of the manufacturer (GE Healthcare). All purified fusion proteins were dialyzed in PBS before use.

### Circular Dichroism Spectrophotometry

Protein samples were dissolved and diluted using 0.01 M HEPES (4-(2-hydroxyethyl)-1-piperazineethanesulfonic acid) buffer (pH 7.4). The samples were allowed to equilibrate to room temperature for 10 min prior to measurements. For ionic strength dependence studies, protein samples were first dissolved in 0.01 M HEPES buffer with 1 mM CaCl_2_ and incubated for 48 h in a temperature controlled VMR incubating Microplate Shaker. CD spectra were recorded with a Jasco J815 spectropolarimeter (Jasco International Co. Ltd., Tokyo, Japan) in a thermostatically controlled 0.1-cm path length cuvette from 190 to 250 nm with 1.00 nm bandwidth and an integration for 1 s at 0.5-nm intervals.

### Surface Plasmon Resonance

Surface Plasmon Resonance (SPR)-based analyses were performed using a Biacore^TM^ X100 SPR system (GE Healthcare, Uppsala, Sweden). Biacore^TM^ sensor chip CM5 was modified with anti-GST antibody (30 μg/mL) via amine coupling using the Amine Coupling Kit (GE Healthcare, Uppsala, Sweden). Both reference channels (channel 1) and sample channels (channel 2) were conditioned with three consecutive injections of 10 mM glycine-HCl (pH 2.1) for 120 s each at a flow rate of 10 μL/min. GST-tagged rLcl proteins (100 nM) were immobilized onto the sample channel for 18 min at a flow rate of 2 μL/min. His-tagged analytes were exposed to the rLcl-modified chip surface at various concentrations (31.25, 62.58, 125, 250, and 500 nM) for 18 min using single-cycle injections at 5 μL/min. The chips were regenerated using 10 mM glycine-HCl (pH 2.1) for 120 s. To determine the full kinetic profile, protein–protein interaction spectrograms were evaluated using Biacore^TM^ X100 Evaluation Software.

### Biofilm Quantification

All biofilm assays were performed using polystyrene 96-well plates (Costar). *L. pneumophila* biofilm assays were performed as previously described (Duncan et al., 2011). Strains were grown for 30 h in BYE and diluted to an OD of 0.2 in fresh broth and incubated for 2 days. Biofilms were stained with 40 μl of 0.25% crystal violet per well for 15 min and washed three times with 200 μl of sterile deionized water. The crystal violet stain was then solubilized in 95% ethanol and after 15 min absorbance was read at 600 nm.

### Size Exclusion Chromatography

Size exclusion chromatography of Lcl was performed using Superdex 200 prep grade resin (Sigma-Aldrich) in a XK16/100 column in PBS using the AKTA Fast Protein Liquid Chromatography (FPLC, GE Healthcare) with a flow rate of 0.5 ml/min. Gel filtration Standards (Bio-Rad) and rLcl were run in triplicate and the molecular weight was estimated from the standard curve based on elution volumes and the molecular weights of the standard proteins.

### Generation of p*lcl Variants* With Different Repeats

To determine the role that Lcl collagenous repeats have in various biological processes, *lcl* was PCR amplified using genomic DNA from clinical isolates using primers 1 and 2 (Table 2). PCR products from clinical isolates LU1536, LR1063, and LR0347 (Table 1) were used to create *lcl* inserts containing 18, 13, and 11 repeats, respectively. Upon PCR amplification of the LU1536 clinical strain, a *lcl* variant was spontaneously produced which contained 14 repeats, which was also transformed into the *lcl* knockout strain. The *lcl* variant containing 2 repeats was synthesized (Genscript) and cloned using the strategy described below. The resulting PCR products and the vector pBH6119 were then digested with *XbaI* and *SphI* to generate compatible ends. The PCR products were then ligated into the *XbaI* and *SphI* digested pBH6119 vector and transformed into *E. coli* TOP10 strain (Invitrogen). Transformants were selected by carbenicillin resistance on LB agar. Single colonies were then picked, cultured in LB broth with 50 μg/ml carbenicillin for plasmid extraction. After verification the plasmid was then transformed into Lp02 and Lp02Δ*lcl* (Table 1).

### SDS-PAGE and Immunoblot Analysis

SDS-PAGE was performed as previously described (Laemmli, 1970). Immunoblotting was performed according to the methods of Towbin et al. (1979). To detect the presence of specific *Legionella* proteins, cell lysates were prepared with plate cultures adjusted to an OD_600__nm_ of 8, centrifuged at 5000 rpm for 10 min and washed twice with PBS. Lysates were then mixed with an equal volume of 2X Laemmli loading buffer with 10% 2-mercaptoethanol, samples were then boiled for 15 min before running on gel. Bound anti-Lcl antibodies (1:20,000) were detected with peroxidase-linked anti-rabbit IgG (1:20,000). Recombinant proteins were detected with anti-His mouse antibody (1:5000) (Invitrogen) and anti-mouse peroxidase linked IgG (1:2000).

### Quantification of *Legionella* Adherence Using Quantitative PCR

Quantitative PCR was performed as previously described (Duncan et al., 2011). To measure the binding abilities of *L. pneumophila* strains to abiotic surfaces, 100 μL of *Legionella* suspension adjusted to an OD_600__nm_ of 2 in PBS was incubated for 1 h at 37°C and 5% CO_2_ in polystyrene 96-well plates (Costar). After three washes with PBS, DNA was purified directly from the wells using a DNeasy 96 blood and tissue kit according to the manufacturer’s instructions (Qiagen). To measure the percent of attached bacteria, DNA was purified from the initial inoculum that was not washed, and percent attached was calculated as the amount of DNA purified from the washed/unwashed wells. Quantitative PCR (qPCR) was performed using primers and a probe to gyrA (Table 2, primers 5–7). Quantitative PCR was performed with Universal PCR master mix (Applied Biosystems) using 400 nM of each primer and 200 nM probe. Amplification and detection was performed with an ABI Prism 7900 detection system. To quantify adherence of bacterial strains to fucoidan the same protocol was followed with 96-well heparin binding plates (BD Biosciences) coated with 5 μgs of fucoidan as per the manufacturer’s recommendations.

### Bacterial Sedimentation Assays

Sedimentation assays were performed as previously described (Abdel-Nour et al., 2013b). To visualize sedimentation, *L. pneumophila* strains were grown for 3 days and colonies were suspended to an OD_600__nm_ of 1 in deionized water with 10% BYE. Images were taken immediately after the indicated time period with all incubations being performed at room temperature. To measure sedimentation kinetics, sedimentation assays were performed as described above, and the OD_600__nm_ was measured at 100 min with a spectrophotometer, where a decrease in OD_600__nm_ indicates an increase in sedimentation. The rate of sedimentation was calculated as the decrease in OD_600__nm_ divided by time.

### Confocal Laser Scanning Microscopy

For confocal laser scanning microscopic examination (CLSM) of biofilms, bacterial cultures (800 μl) were prepared in Lab-TekII chamber slides (Labtek II, VWR, Rochester, NY, United States) according to the procedure described above. After 3 days of incubation at 37°C and 5% CO_2_, 400 μL of supernatant was removed and bacteria were labeled with nucleic acid stain SYTO 62 (Molecular probes) diluted 1:25 for 1 h at RT. Afterwards 400 μL of supernatant was removed and 8% PFA was added for 20 min followed by two washes with sterile deionized water. The plastic wells were removed from the slide and fluoromount (DAKO North America INC., Carpinteria, CA, United States) was added before placing a coverslip on the gasket and observed by CLSM using a Nikon Eclipse TE2000EZ inverted microscope, 100× Plan APO oil immersion DIC N2 objective. Image acquisition and post-acquisition processing were performed using EZ-C1 Software Ver. 3.50 and the NIS-elements BR Software Ver. 3.0 for Nikon C1 Confocal Microscopy.

### Determination of *lcl* Repeat Number in *L. pneumophila* Clinical Isolates

To measure the approximate size of *lcl* in clinical isolates primers 1 and 2 were used (Table 3), and the PCR product was compared to a 2log ladder (Fermentas). To estimate the number of repeats in *lcl* from clinical isolates, chromosomal DNA was amplified with Taq DNA polymerase (Invitrogen) using primers 3 and 4 (Table 2). The PCR product was then compared against a 100 base pair DNA ladder (Fermentas) on a 3% agarose gel. 187 *L. pneumophila* clinical strains from Ontario, Canada from the Public Health Ontario repository (Tijet et al., 2010) and 280 clinical isolates from the French National Reference Center for *Legionella* (Cassier et al., 2015).

**Table 3:** Primers and probes used in this study.

Code	Primer/probe	Amplification target	Sequence 5′–3′
1	*lcl* Xba1 F	*lcl*	GGTCATCTAGAGAAATAAAGAATGATACATCGA
2	*lcl* Sph1 R	*lcl*	GTGAGCGCATGCGCAAAGCGAATTTATGAACA
3	Lpms31 L	*lcl* repeats	GCAATCCGGCCTCGCAAGCC
4	Lpms31 R	*lcl* repeats	CAGGCACACCTTGGCCGTCA
5	*gyrA* F	*gyr*A	GGCGGGCAAGGTGTTATTT
6	*gyrA* R	*gyr*A	GCAAGGAGCGGACCACTTT
7	*gyrA* probe	*gyr*A	VIC-CATTTCGTTCGTAACCTG-MGBNFQ
8	*lcl* Xho1 F	*lcl*	AGCTCGAGCAATCCGGCCTCGCAAGCC
9	*lcl EcoR1* R	*lcl*	CGGAATTCCGGGTTGCGAGAGTTGGCTA

### Graphs and Statistics

Data were plotted and analyzed in GraphPad Prism 5, using the one-way ANOVA analysis tool, all experiments performed in triplicate.

## Author Contributions

MA-N, MT, KK, SJ, and CGu conceived and designed the experiments. MA-N, HS, CD, SL, FS, CGi, DR, and MV performed the experiments and analyzed the data. MA-N, CGu, KK, and MT wrote the manuscript.

## Conflict of Interest Statement

The authors declare that the research was conducted in the absence of any commercial or financial relationships that could be construed as a potential conflict of interest.
