# Supplementary material for: Polymorphisms of a Collagen-Like Adhesin Contributes to Legionella pneumophila Adhesion, Biofilm Formation Capacity and Clinical Prevalence
Source: Front Microbiol. 2019 Apr 5;10:604. doi: 10.3389/fmicb.2019.00604 (PMC6460258; doi:10.3389/fmicb.2019.00604)
Supplement: Supplementary file 1 [file Table_1.DOCX]

**Supplementary Tables**

**Table S1. Comparison of predicted amino acid sequences in Lcl isoforms**

| Sequences being compared | Origin of sequence | Shared Identity (amino acid) |
| --- | --- | --- |
| Lcl 18repeats - Lcl 13repeats | LU1536-LR1063 | 95% |
| Lcl 18repeats - Lcl 14repeats | LU1536-LU1536 | 100% |
| Lcl 18repeats - Lcl 11repeats | LU1536- LR0347 | 94.9% |
| Lcl 13repeats – Lcl 11repeats | LR1063-LR0347 | 99.5% |

**Supplementary Figure Legends**

**Figure S1. Lcl isoforms and growth of the *L. pneumphila* clinical isolates in this study.** (A)Schematic representation of the different Lcl isoforms used in this study. The light grey boxes represent the N terminal signal sequence, the dark grey boxes represent the tandem repeats comprised of five Gly-Xaa-Yaa tripeptides each (15 amino acids per repeat) and the black boxes represent the C terminal domain. The Lcl variant containing 18 repeats was found in the LU1536 clinical strain and is identical to the isoform found in the Lp02 wild-type strain. The Lcl variant containing 14 repeats was obtained as a spontaneous PCR product after amplification of LU1536 *lcl.* Lcl containing 13 and 11 repeats was obtained from the clinical isolates LR1063 and LR0347 respectively. (B) Growth of the *L. pneumophila* clinical strains used in this study measured by OD600nm absorbance measurements of 2day old biofilms after disruption by vigorous pipetting.

**Figure S2. The *L. pneumophila* isogenic strains in this study.** (A) Anti-Lcl Immunoblots of whole cell lysate from the indicated bacterial strains, NS* denotes a nonspecific band. (B) Growth of the *L. pneumophila* isogenic strains used in this study measured by OD600nm absorbance measurements of 2day old biofilms after disruption by vigorous pipetting.
