# Supplementary figures and images for: Polymorphisms of a Collagen-Like Adhesin Contributes to Legionella pneumophila Adhesion, Biofilm Formation Capacity and Clinical Prevalence
Source: Front Microbiol. 2019 Apr 5;10:604. doi: 10.3389/fmicb.2019.00604 (PMC6460258; doi:10.3389/fmicb.2019.00604)

A

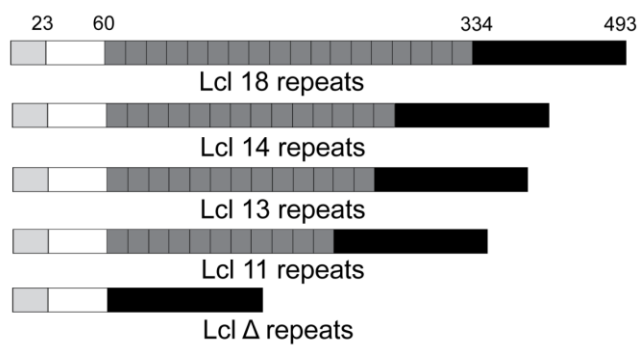

B

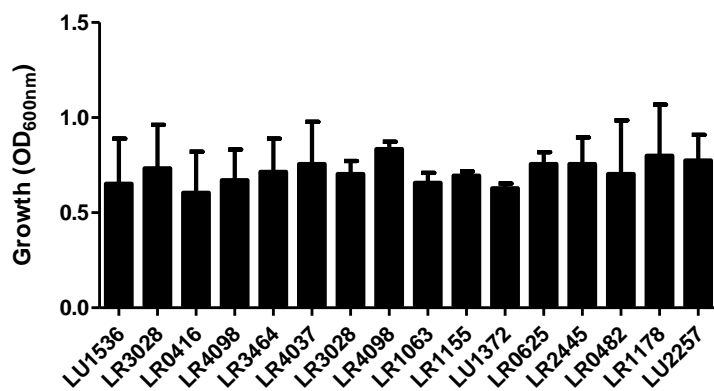

A

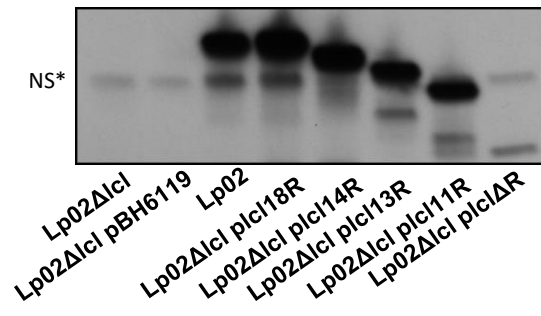

B

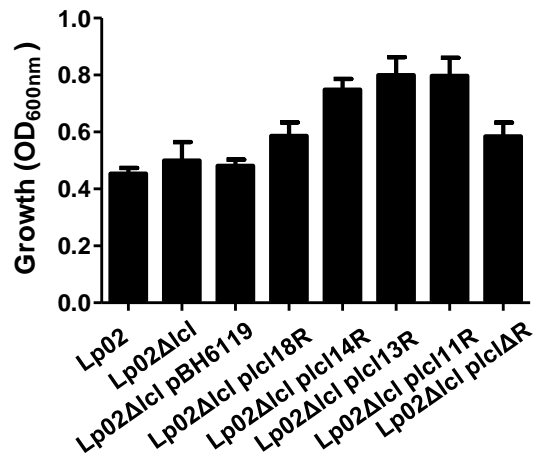

Supplement: Supplementary file 2 [file Data_Sheet_1.PDF]
